# Validation of *Eustiromastix guianae* (Caporiacco, 1954) (Araneae, Salticidae) with a first description of the female, and additions to the salticid fauna of French Guiana

**DOI:** 10.3897/zookeys.420.6977

**Published:** 2014-06-25

**Authors:** Cyril Courtial, Lionel Picard, Frédéric Ysnel, Julien Pétillon

**Affiliations:** 1 6 Avenue Pierre Donzelot 34B 35700 Rennes, France; 2 13 rue Jean-Baptiste Carpeaux 56000 Vannes, France; 3 Equipe Biodiversité et Gestion des Territoires URU 420, UFR SVE, Université de Rennes I, 263 Avenue du Général Leclerc 35042 Rennes Cedex, France

**Keywords:** Jumping spider, tropical forest, South America, doubtful species

## Abstract

In this paper, we validate the doubtful species status of *E. guianae*, with redescriptions of (supposedly lost) type and holotype males, and a first description of the female. Both sexes are measured and illustrated by pictures of habitus and copulatory organs. Seventeen new salticid species for French Guiana are also reported and a detailed catalogue of all salticid species from the Trinité National Nature Reserve is provided.

## Introduction

The history of arachnology in French Guiana started in 1871 with the publication of the first catalogue by Władysław Taczanowski (1871, 1872), but it remained relatively poor compared with that of adjacent countries (Brazil, Guiana, Peru, etc.). It reached its apogee in the middle of the 20^th^ century with the work of Di Caporiacco (1954), which clearly pleads for an urgent update on this group. The Salticidae is one of the most important spider families, with 597 genera and nearly 5700 described species worldwide (Platnick 2014) which are particularly abundant and diversified in the neotropical region (Dias et al. 2006), yet this family is poorly known in French Guiana with only 85 reported species (Vedel et al. 2013) compared to the 539 species known in Brasil (Metzner 2014). During a recent survey conducted in the Trinité National Nature Reserve (French Guiana) we collected numerous species of salticidae and among them several specimens of the genus *Eustiromastix* Simon, 1902. The spider genus *Eustiromastix* has 11 species distributed throughout South America and the southern West Indies according to Platnick (2014). Among this genus, *Eustiromastix guianae* (Caporiacco, 1954) was considered *nomen dubium* by Galiano (1979) because 1) the type was lost and not viewed 2) morphological details referring to the palp and the ambulatory formula do not fit the description of the genus.

Based on the rediscovery of the type specimen in the collection of the MNHN of Paris and on the collection of several males and females, we propose to validate the doubtful species *Eustiromastix guianae* here, and provide the first description of the female. In addition we provide a detailed catalogue of all salticid species from the Trinité National Nature Reserve, with new species for French Guiana after Vedel et al. (2013).

## Material and methods

### Description

The following abbreviations are used:

AER anterior eye row; ALE anterior lateral eyes; AME anterior median eyes; PER posterior eye row; PLE posterior lateral eyes; PME posterior median eyes; NNR National Nature Reserve.

All the fresh specimens examined in this study were collected on leaves of several tropical trees during a survey at the Trinité NNR in December 2010. Measurements (in millimetres as in Galiano 1963) were taken on four males (the holotype and three other males) and on seven females. The leg spination was not assessed on the holotype, but on a fresh specimen. The specimens were studied using a Euromex CMEX 5000 stereomicroscope. The epigyne was macerated in 10% KOH. The specimens were preserved in 70% ethanol.

### Catalogue of the National Reserve

Regional literature on salticids was consulted and the presence of valid species after Platnick (2014) was established. New data from the survey were included as well as updates. For all species, the date of first publication, locality, information about sex and dates of collection are given in Suppl. material 1 (Table 1).

## Results

### Description

#### Eustiromastix guianae

Taxon classificationAnimaliaAraneaeSalticidae

(Caporiacco, 1954)

Eustiromastix guianae (Caporiacco, 1954): 176–177, figure 65, 65a; Galiano, 1979: 185.

##### Material.

Holotype: male (MNHN): French Guiana, Charvein. 7 females, 3 males and 1 subadult male Trinité NNR, 04°36'02"N, 53°24'43"W, 09.XII.2010, Julien Pétillon and Cyril Courtial. Specimens have been deposited in the Museum National d’Histoire Naturelle, Paris (male collection number: AR 15000; female collection number: AR 15001).

##### Diagnosis.

Among salticids, the differenciation between genera of the Plexippeae group is especially complex (Galiano 1979) and only based on genitalia. *Eustiromastix* and *Freya* are close relatives. *Freya* is distinguished by its short thick pedipalp and tibial apophyses often rectangular and wide, and the solid and robust embolus, while *Eustiromastix* presents a longer pedipalp with a very long thin embolus (Galiano 1979) and an apically curved cymbium. Females are characterised by the wide, ﬂattened and folded copulatory ducts, which are as wide as the spermathecae (Santos and Romero 2004). *Eustiromastix guianae* differs from all species of the genus by the shape of the embolus, the presence of a long median apophysis and the small pointed tibial apophysis. The female is distinguished by the strong vertical ducts and the large openings on the epigyne.

##### Redescription of the male

**Figure 1.**
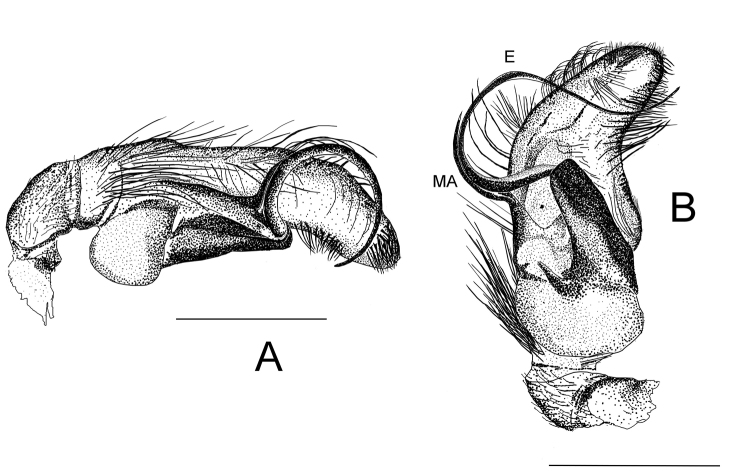
Drawing of the Holotype of *Eustiromastix guianae* male palp (**A** prolateral view **B** ditto ventral view) **MA**: median apophysis, **E**: embolus. Scale 1 mm.

**Figure 2.**
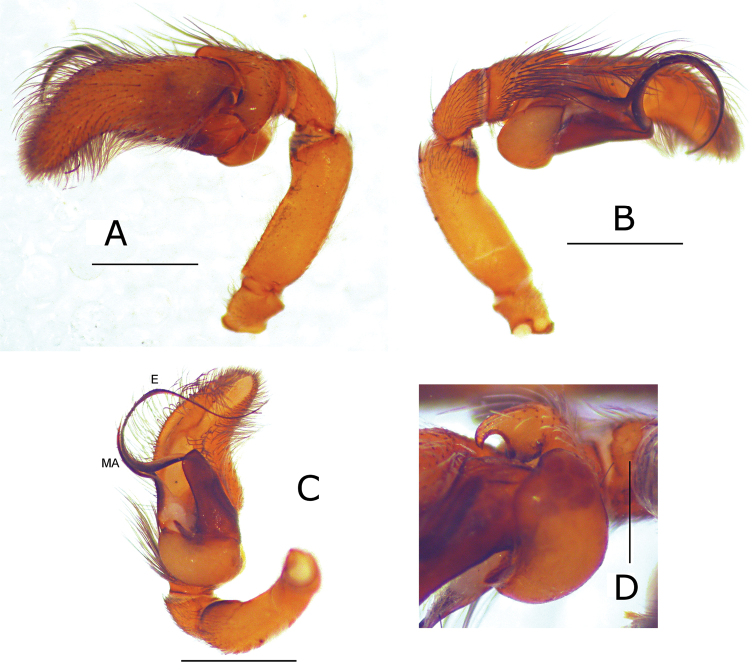
Pictures of the male palp of *Eustiromastix guianae*. **A–C** male palp in lateral, retrolateral and ventral views, respectively **D** detail of the tibial apophysis, ventral view. **MA** median apophysis, **E** embolus. Scales: **A–C** 1 mm, **D** 0.5 mm.

**(holotype from Charvein).** Figs 1A, B; 2A–D; 5A. Total length 6.54. Prosoma: carapace 2.95 long, 2.35 wide and 1.83 high. Carapace: darkish brown. Cephalic region: black, and darker than thoracic region. Diameter of PME: 0.34; PLE: 0.14; AME: 0.58; ALE: 0.46. Distance PLE-PLE: 1.94; PME-PME: 2.01; PME-PLE: 0.27. Chelicerae: 1.10 long and 0.77 wide, dark brown. One promarginal and two retromarginal teeth. Legs: light brown, with dark annulations. Leg formula 3412. Length of femur: I 2.09, II 1.84, III 2.55, IV 2.27; patella: I 1.29, II 1.04, III 1.17, IV 0.87; tibia: I 1.80, II 1.32, III 1.42, IV 1.40; metatarsi: I 1.26, II 1.10, III 1.70, IV 1.83; tarse: I 0.66, II 0.62, III 0.61, IV 0.70.

Spination: femur I-III d1-1-1, r2-2 p2-2, IV d1-1-1, r1; patella I-II p1, III-IV p1, r1; tibia I-II v2-2-2, III-IV v1-2, p3, r3, d1; metatarsi I-II v2-2, III-IV v2-2, p1, r1, d2-2-2. Abdomen 3.71 long.

Dorsum: greyish with diffuse pale spots, and a median, longitudinal light brown stripe between a pair of longitudinal narrow stripes of white scales anteriorly and a white chevron posteriorly in fresh specimens (see Fig. 5A). Abdominal pattern with a median dark brown band.

Male palp as in Fig. 1A, B. Cymbium flattened apically (Fig. 2A), small retrolateral tibial apophysis, pointed internally in dorsal view (Fig. 2D). Long embolus with an elongated median apophysis at about one third of the total length of the embolus (Fig. 2B, C).

Locality: Charvein.

##### Female.

**Figure 3.**
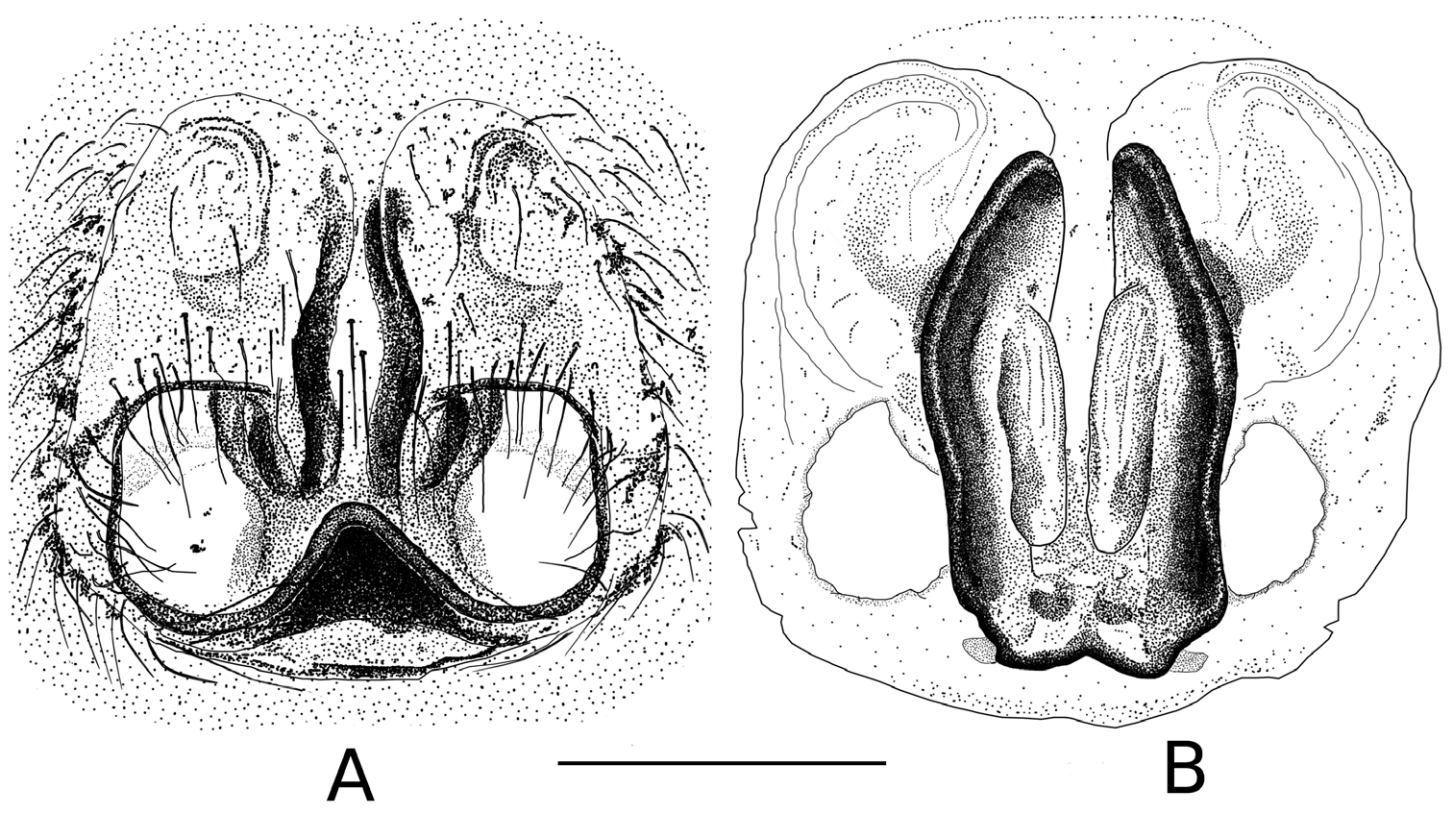
Drawing of *Eustiromastix guianae* epigyne (**A** dorsal view **B** ventral view). Scale 0.5 mm.

**Figure 4.**
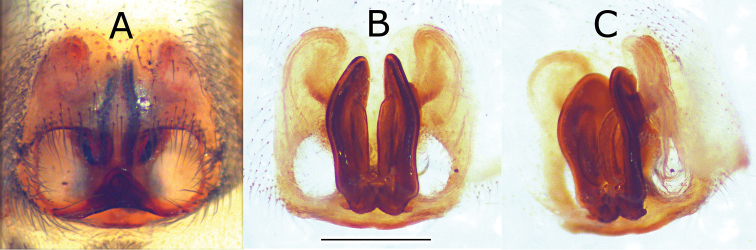
Pictures of the epigyne and spermathecae of *Eustiromastix guianae*. **A** dorsal view **B** vulva, ventral view after maceration **C** ditto latero dorsal view. Scale: 0.5 mm.

**Figure 5.**
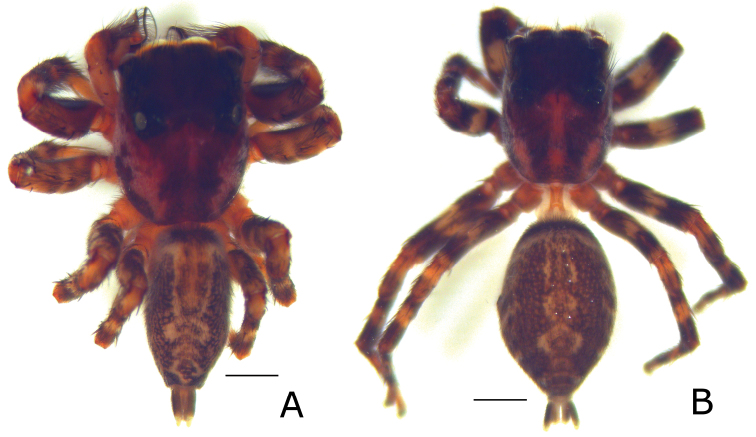
Habitus of *Eustiromastix guianae*, male, fresh specimen (**A**) and female (**B**). Scale: 1 mm.

Figs 3A, B; 4A–C; 5B. Total length: 7.51. Prosoma: carapace 2.75 long, 2.19 wide, and 1.59 high. Carapace: dark brown. Cephalic region: darker than the thoracic region. Diameter of PME: 0.29; PLE: 0.10; AME: 0.62; ALE: 0.32. Distance PLE-PLE: 1.75; PME-PME: 1.83; PME-PLE: 0.30. Chelicerae: 0.83 long 0.49 wide. Legs formula 4312. Length of femur: I 1.66, II 1.52, III 1.85, IV 1.89; patella: I 0.80, II 0.69, III 0.85, IV 0.71; Tibia: I 1.29, II 1.08, III 1.13, IV 1.30; metatarsi: I 0.74, II 0.64, III 1.16, IV 1.46; tarsi: I 0.62, II 0.58, III 0.67, IV 0.67. Spination: femur I-II d1-1-1, p2, III d1-1-1, p1, r1, IV d1-1-1, r1; patella: I-II p1, III-IV r1; tibia: I-II v2-2-2, p1, III-IV v1-2, r1-1-1, d1, p1-1-1; metatarsi: I-II v2-2, III d2-1-2, r1, v2-2, IV d2-2, v2-2, r1. Abdomen: 3.66 long. Dorsum: greyish to blackish (Fig. 5B) with a diffuse pale chevron in median part and a small white spot lateraly. Epigyne and spermathecae: as in Fig. 3A, B. Epigyne with two deep circular genital openings (Figs 3A, 4A). Insemination ducts: long and parallel (Figs 3B, 4B, C). Spermathecae: almost round and small at the base of the vulva (Fig. 4B, C).

##### Variation.

**Male**: Total length: 6.50–7.17, carapace length: 2.40–3.38, carapace width: 2.26–2.66 (n=4). **Female**: total length: 6.32–7.51, carapace length: 2.75–3.11, carapace width: 2.19–2.31 (n=7).

##### Habitat.

all specimens were collected together, including males in couple with females in tropical rain forest from beating and sweeping low vegetation.

##### Distribution.

known only from two localities in French Guiana (Charvein and Trinité NNR) Figure 1 of Suppl. material 1.

### Addition to the checklist of Salticid species known from French Guiana

Twenty-eight jumping spiders have been identified at the Trinité National Nature Reserve, among them 17 are new for French Guiana (see the catalogue of saticids of the reserve: Table 1 of Suppl. material 1). A total of 102 salticid species is now recorded from French Guiana.

## Supplementary Material

XML Treatment for Eustiromastix guianae

## Supplementary material

Supplementary material 1 Electronic supplementary material doi: 10.3897/zookeys.420.6977.app1 Data type: Specimens data Explanation note: Table 1. Detailed records of all salticid species in the National Nature Reserve Trinité. Figure 1. Map showing localities of NNR Trinité and Charvein. This dataset is made available under the Open Database License (http://opendatacommons.org/licenses/odbl/1.0/). The Open Database License (ODbL) is a license agreement intended to allow users to freely share, modify, and use this Dataset while maintaining this same freedom for others, provided that the original source and author(s) are credited.Cyril Courtial, Lionel Picard, Frederic Ysnel, Julien Pétillon

## References

[B1] Caporiacco L Di (1954) Araignées de la Guyane Française du Museum d’Histoire Naturelle de Paris. Commentationes - Pontificia Accademia Scientiarum 16: 45–193

[B2] Dias SC Brescovit AD Couto ECG Martins CF (2006) Species richness and seasonality of spiders (Arachnida, Araneae) in an urban Atlantic forest fragment in Northeastern Brazil. Urban Ecosystems 9: 323–335. doi: 10.1007/s11252-006-0002-7

[B3] Galiano ME (1963) Las especies americanas de aranas de la familia Salticidae descriptas por Eugene Simon: Redescripciones basadas en los ejemplares tipicos. Physis B. Aires (C) 23: 273–470

[B4] Galiano ME (1979) Revision del genero *Eustiromastix* Simon, 1902 (Araneae, Salticidae). Journal of Arachnology 7: 169–186

[B5] Metzner H (2014) Worldwide database of jumping spiders (Arachnida, Araneae, Salticidae). http://www.jumping-spiders.com/[accessed 23.IV.2014]

[B6] Platnick NI (2014) The world spider catalog, version 13. American Museum of Natural History. http://research.amnh.org/entomology/spiders/catalog/index.html [accessed 13.XI.2012]

[B7] Santos AJ Romero GQ (2004) A new Bromelliad-dwelling jumping spider (Araneae, Salticidae) from Brazil. Journal of Arachnology 32: 188–190. doi: 10.1636/H03-01

[B8] Taczanowski L (1871) Les Aranéides de la Guyane française. Horae Societatis Entomologicae Rossica 8: 32–132. [pl. III–IV]

[B9] Taczanowski L (1872) Les aranéides de la Guyane française. Horae Societatis Entomologicae Rossica 9: 64–112

[B10] Vedel V Rheims C Murienne J Brescovit AD (2013) Biodiversity baseline of the French Guiana spider fauna. SpringerPlus 2: 1–19. doi: 10.1186/2193-1801-2-361 23961423 10.1186/2193-1801-2-361 PMC3738911

