# Supplementary material for: Validation of Eustiromastix guianae (Caporiacco, 1954) (Araneae, Salticidae) with a first description of the female, and additions to the salticid fauna of French Guiana
Source: Zookeys. 2014 Jun 25;(420):11–8. doi: 10.3897/zookeys.420.6977 (PMC4109477; doi:10.3897/zookeys.420.6977)
Supplement: Supplementary material 1 — Electronic supplementary material [file zookeys-420-011-s001.pdf]

Electronic supplementary material

Table 1: Detailed records of all salticid species in the National Nature Reserve Trinité (Date and locality: date (year, or present paper: PP) and locality of the first published record for French Guiana the region; Abbreviations: NNR National Nature Reserve, Aya Tropical rainforest site « Aya », **RB** « Roche Bénitier » – Inselberg; *** only known from French Guiana; bold: new species for French Guiana).

Species	Date	Locality	Additional data
***Amatorculus cristinae* Ruiz & Brescovit, 2006**	PP	NNR Trinité	1♀: 14.XII.2010 (Aya)
***Amycus pertyi* Simon, 1900**	PP	NNR Trinité	2♂, 1♀: 31.X.2008; 1♂: 01.XI.2008; 5♂, 2♀: 09.XII.2010 (Aya)
Chinoscopus maculipes Crane, 1943	2013	NNR Trinité	1♀: 09.XII.2010 (Aya).
***Eustiromastix falcatus* Galiano, 1981**	PP	NNR Trinité	1♂: 09.XII.2010 (RB), 1♂: 15.XII.2010 (Aya)
Eustiromastix major Simon, 1902	2013	NNR Trinité	1♀: 26.X.2008 (Aya), 1♀: 15.XII.2010 (RB)
***Hypaeus miles* Simon, 1900**	PP	NNR Trinité	1♀: 01.XI.2008 (Aya), 1♂, 1♀: 15.XII.2010 (Aya)
Hypaeus porcatus (Taczanowski, 1871)***	1871	Saint Laurent	1♂: 08.XII.2010; 1♂: 09.XII.2010 (Aya); 1♂:

		du Maroni	15.XII.2010 (RB)
Hypaeus taczanowskii (Mello-Leitão, 1948)	1954	Charvein, Saint Jean du Maroni Maroni	6♂, 4♀: 15.XII.2010 (RB); 2♂, 2♀: 01.XI.2008; 6♂, 6♀: 09.XII.2010 (Aya)
Kalcerrytus nauticus Galiano, 1999	2013	NNR Trinité	1♂: 15.XII.2010 (RB)
***Lyssomanes benderi* Logunov, 2002**	PP	NNR Trinité	1♂: 09.XII.2010 (Aya); 1♀: 15.XII.2010 (RB)
Lyssomanes ipanemae Galiano, 1980	2013	NNR Trinité	1♂: 25.X.2008 (Aya)
***Lyssomanes jemineus* Peckham & Wheeler, 1888**	PP	RNN Trinité	1♀: 25.X.2008 (Aya)
Lyssomanes longipes (Tacznowski, 1872)	1872	Saint Laurent du Maroni	2♂, 8♀: 09.XII.2010 (Aya); 6♂, 8♀: 15.XII.2010 (RB)
***Lyssomanes nigropictus* Peckham & Wheeler, 1889**	PP	NNR Trinité	1♀: 09.XII. 2010 (Aya)
Mago longidens Simon, 1900	1954	Sault Mouciri	2♂, 7♀: 09.XII.2010 (Aya); 1♂, 4♀: 26.X.2010 (Aya); 3♂, 2♀: 15.XII.2010 (RB)
Mago silvae Crane, 1943	1954	Charvein	2♂, 2♀: 09.XII.2010 (Aya); 2♂, 4♀: 15.XII.2010 (RB)

***Noegus franganilloi* (Caporiacco, 1947)**	PP	NNR Trinité	1♂: 09.XII.2010 (Aya)
***Noegus niveomarginatus* Simon, 1900**	PP	NNR Trinité	3♂, 1♀: 09.XII.2010 (Aya); 1♂, 1♀: 15.XII.2010 (RB)
***Noegus transversalis* Simon, 1900**	PP	NNR Trinité	2♂:26.X.2008; 1♂: 09.XII.2010 (Aya)
***Pachomius sextus* Galiano, 1994**	PP	NNR Trinité	1♂: 15.XII.2010 (RB)
Phiale crocea C. L. Koch, 1846	2013	NNR Trinité	1♂: 09.XII.2010 (Aya)
***Romitia nigra* (Caporiacco, 1947)**	PP	NNR Trinité	1♂: 09.XII.2010 (Aya)
***Soesilarishius aurifrons* (Taczanowski, 1878)**	PP	NNR Trinité	2♂: 09.XII.2010 (Aya)
***Soesilarishius ruizi* Zhang & Maddison, 2012**	PP	NNR Trinité	1♀: 15.XII.2012 (RB)
***Synemosyna paraensis* Galiano, 1967**	PP	NNR Trinité	1♀: 09.XII.2010 (Aya); 1♀: 15.XII.2010 (RB)
***Thiodina pseustes* Chamberlin & Ivie, 1936**	PP	NNR Trinité	1♀: 21.X.2008 (Aya)
***Zuniga magna* Peckham & Peckham, 1892**	PP	NNR Trinité	2♂: 09.XII.2010 (Aya); 3♀: 15.XII.2010 (RB)

Figure 1. Pictures of the male palp of *Eustiromastix guianae*. A – C: male palp in lateral, retrolateral and ventral views, respectively. D: detail of the tibial apophysis, ventral view. Scales: A - C 1mm, D 0.5mm.

Figure 2. Pictures of the epigyne and spermathecae of *Eustiromastix guianae*. A: dorsal view; B: vulva, ventral view after maceration; C: ditto latero dorsal view. Scale: 0.5 mm.

Figure 3. Habitus of *Eustiromastix guianae*, male, fresh specimen (A) and female paratype (B). Scale: 1 mm.

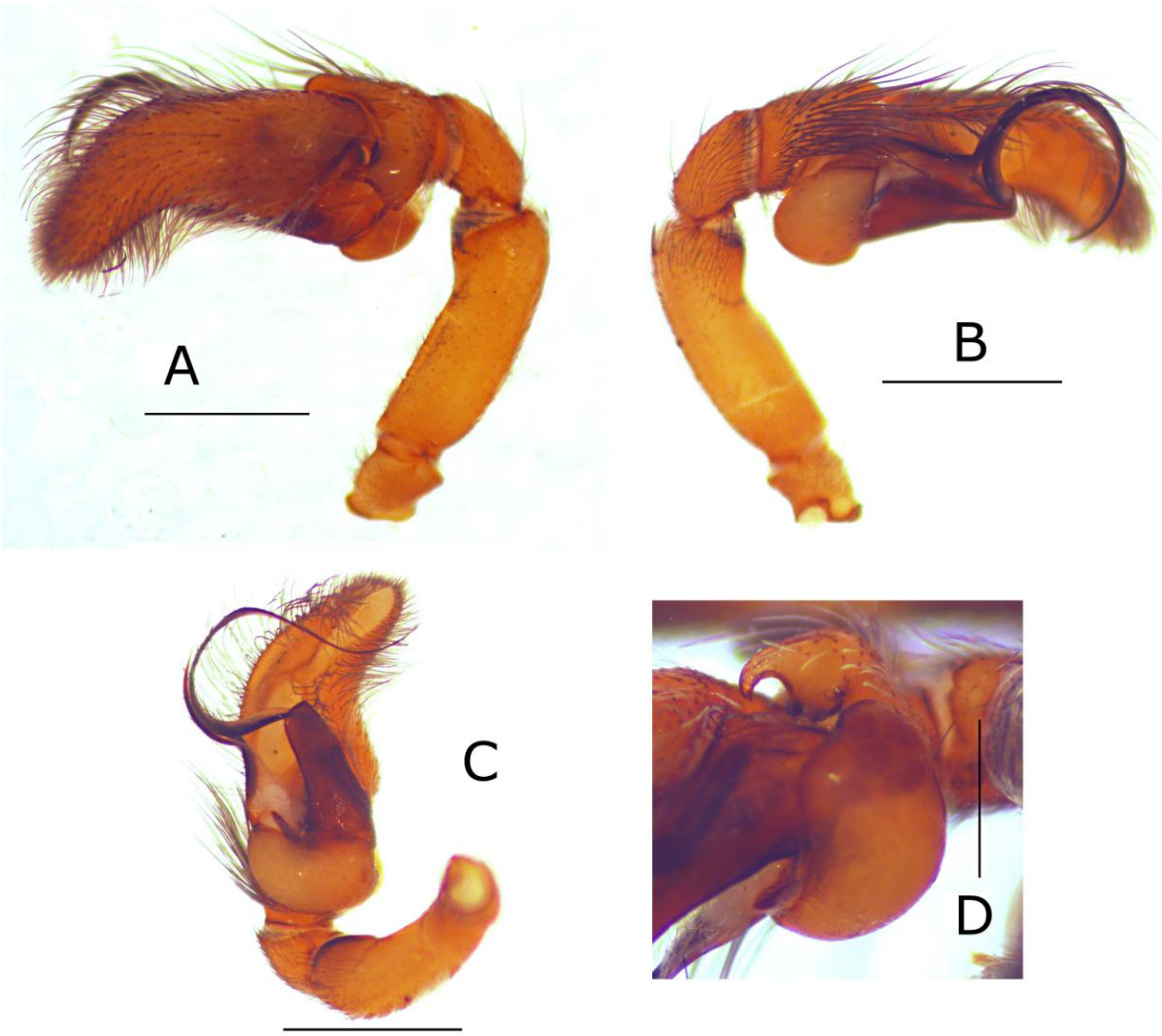

Figure 1 A - D

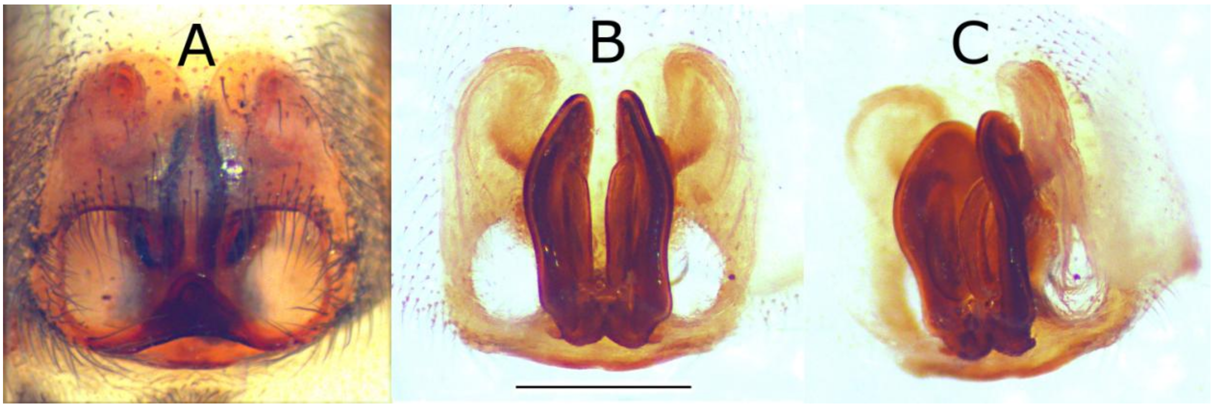

Figure 2 A - C

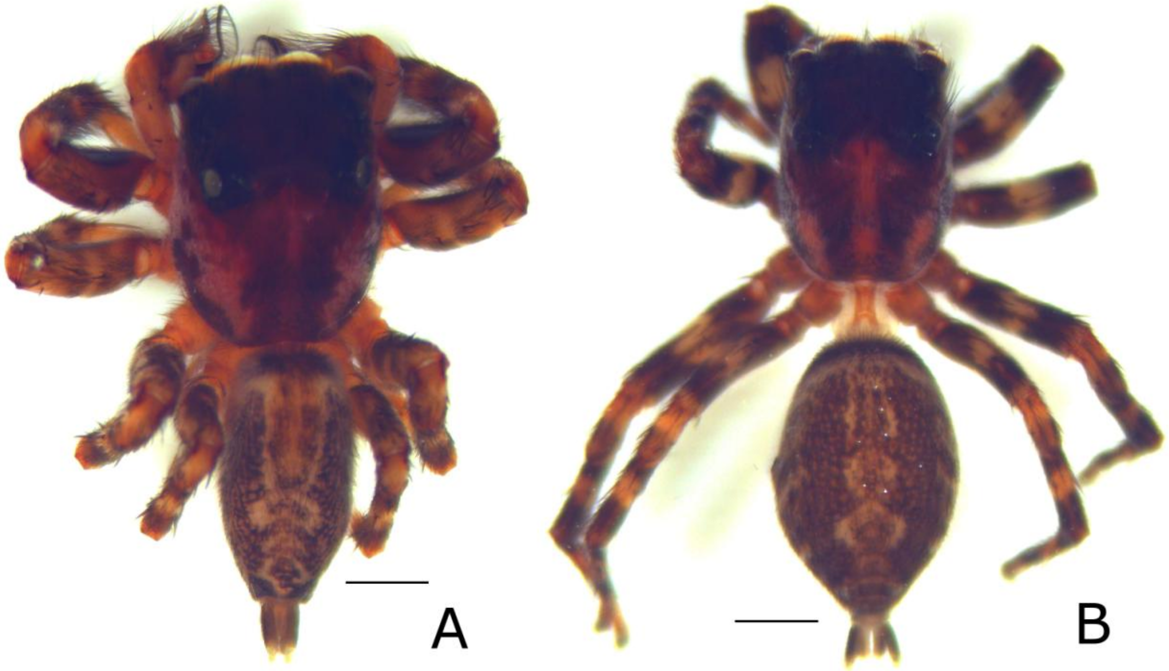

Figure 3 A, B
